# P-871. Can We Change What We Do Not Measure? Antibiotic Prescribing Patterns in Wisconsin’s Urgent Care Centers in 2022

**DOI:** 10.1093/ofid/ofaf695.1079

**Published:** 2026-01-11

**Authors:** Sura AlMahasis, James H Ford, David Mott, Lindsay Taylor

**Affiliations:** School of Pharmacy, University of Wisconsin-Madison, MADISON, WI; University of Wisconsin-Madison, Madison, Wisconsin; Division of Social and Administrative Sciences, University of Wisconsin-Madison, Madison, Madison, Wisconsin; University of Wisconsin School of Medicine and Public Health, Madison, Wisconsin

## Abstract

**Background:**

Urgent care centers (UCCs) are a fast-growing outpatient healthcare model due to their convenience and affordability; however, most UCCs lack formal antimicrobial stewardship (AMS) programs. Characterizing antibiotic prescribing is essential for identifying improvement targets. This study examined 2022 antibiotic prescribing patterns in Wisconsin’s UCCs to inform future AMS efforts.

**Figure:**
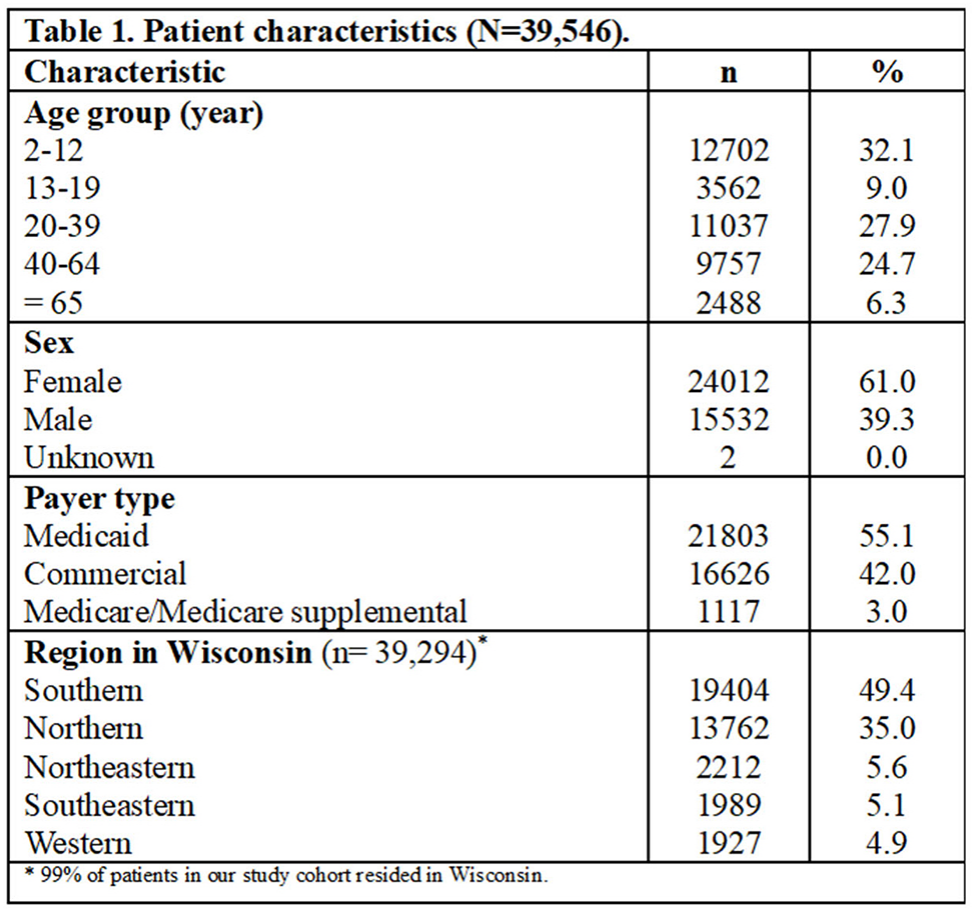

**Figure:**
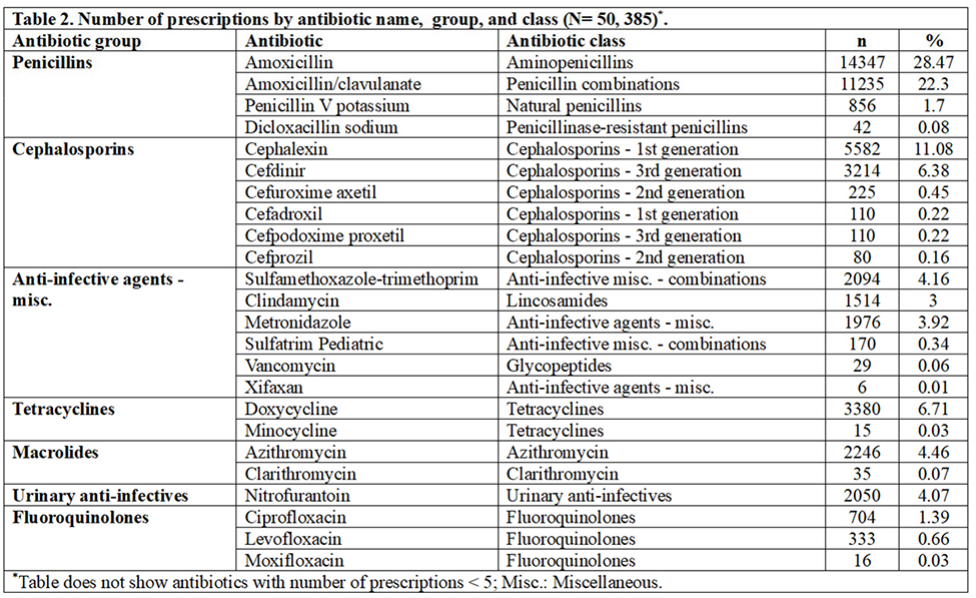

**Methods:**

We conducted a retrospective analysis of 2022 antibiotic prescriptions at UCCs using all-payer claims data from the Wisconsin Health Information Organization. Our cohort included patients who filled ≥ 1 oral antibiotic prescription on or within 3 days of a UCC visit. ICD-10 diagnosis codes were used to identify the indications and grouped into antibiotic appropriateness tiers. Patient characteristics included sex, age, payer type, and region. We used the prescriber national provider identifier (NPI) taxonomy to categorize provider types. Data were curated and analyzed in SAS 9.4 using descriptive statistics.

**Figure:**
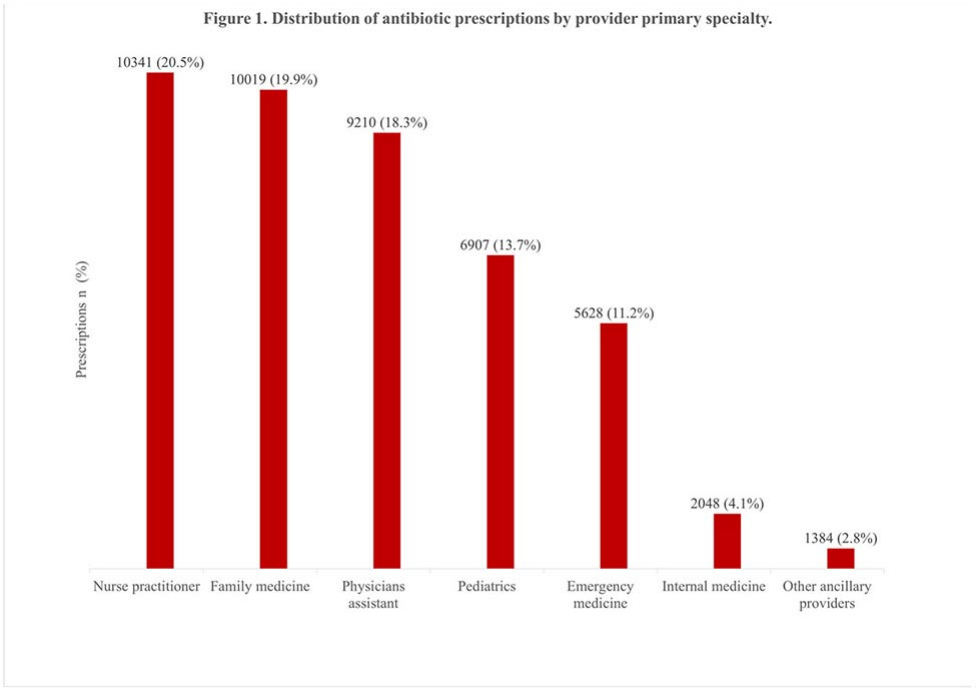

**Figure:**
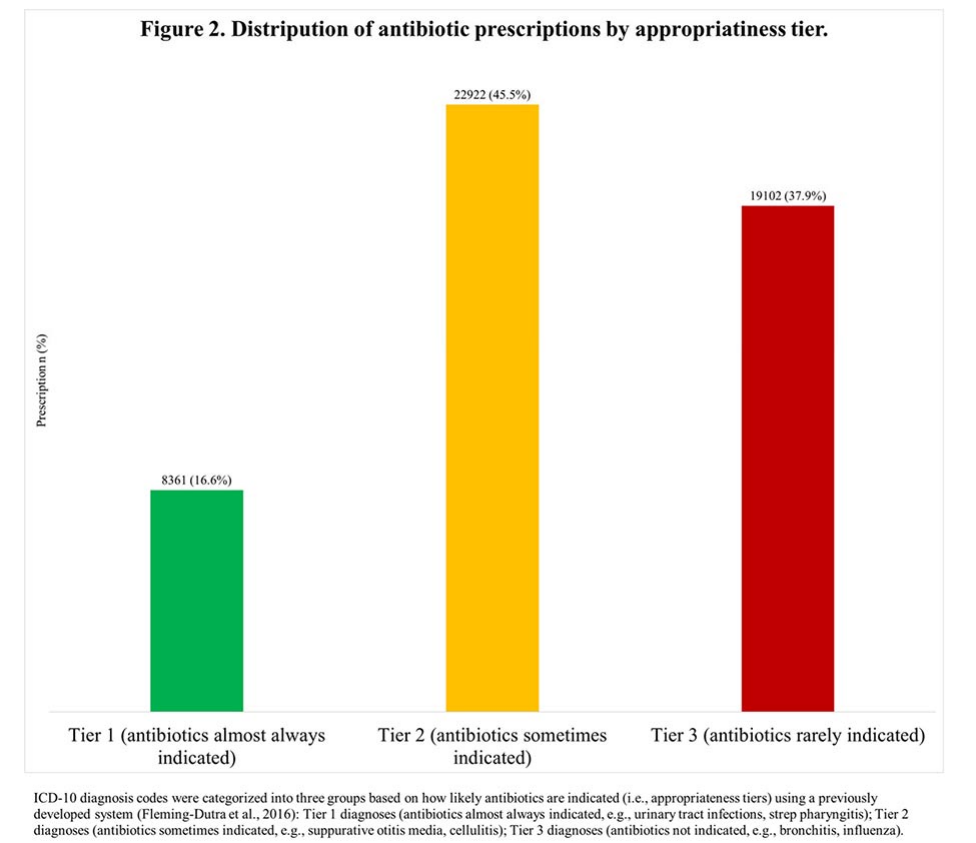

**Results:**

The study cohort included 39,546 patients who collectively filled 50,385 antibiotic prescriptions after being seen at one of 388 UCCs in Wisconsin. Of the patients, 61.0% were female, 32.1% were aged 2-12, 55.1% were insured by Medicaid, and nearly half lived in the southern region of Wisconsin (Table 1). The median number of prescriptions per UCC was 24 (IQR: 4.0-87.0), and just 10% of providers (303/3,027) accounted for around 80.0% of all antibiotic prescriptions (n= 40,169). Nurse practitioners prescribed the most (20.5%), followed by family medicine physicians (19.9%) and physician assistants (Figure 1). Over 70.0% of prescriptions were for 5 antibiotics: amoxicillin (28.5%), amoxicillin/clavulanate (22.3%), cephalexin (11.1%), cefdinir (6.4%), and azithromycin (4.5%) (Table 2). The 3 most common associated conditions were otitis media (12.3%), acute pharyngitis (4.7%), and acute sinusitis (3.8%). Nearly 40% of prescriptions were for Tier 3 conditions (Figure 2).

**Conclusion:**

A minority of UCC providers prescribed the majority of antibiotics, suggesting targeted provider feedback may be a successful stewardship intervention in Wisconsin UCCs. Future studies should incorporate additional prescribing metrics to further guide AMS efforts in UCCs.

**Disclosures:**

All Authors: No reported disclosures

